# Supplementary material for: An analysis of adolescent leisure activity structure based on subjective well-being: Focusing on social network analysis
Source: PLoS One. 2025 May 9;20(5):e0322956. doi: 10.1371/journal.pone.0322956 (PMC12064206; doi:10.1371/journal.pone.0322956)
Supplement: Attachment — Submitted filename: Response to Reviewers5.docx [file pone.0322956.s002.docx]

To the Editors and Reviewers of PLOS ONE,

We sincerely appreciate your review and valuable feedback on our study. We have carefully considered the comments from Reviewer 5 and have incorporated several revisions to enhance the scientific rigor and clarity of our manuscript. Below, we provide our responses to the key points raised by the reviewer and outline our revision plan.

**[Reviewer 5]**

Congratulations on bringing your research to this stage. We appreciate your efforts in sharing your findings. However, the following key aspects need to be addressed:

1. Clearly explain the theoretical and conceptual framework that guided the study. Add two paragraphs explaining why SNA is the most appropriate methodology for this study.

- Thank you very much for your insightful comments. We have added two paragraphs in the Introduction section to explain why SNA is the most suitable methodology for this study. (115~134 lines on page 5).

2. Clearly state the research questions and hypotheses.

- Thank you very much for your insightful comments. We have revised and supplemented the end of the Introduction section to clearly present the research questions and hypotheses. (153~164 lines on page 6).

3. Discuss whether the 2019 dataset remains valid in the current context.

- Thank you very much for your insightful comments. We have added a clear explanation regarding the validity of the 2019 dataset in the present context in the Methods – Study Participants section. (242~261 lines on page 8).
